# The application of a novel integrated rigid and flexible Nephroscope in percutaneous nephrolithotomy for renal staghorn stones

**DOI:** 10.1186/s12894-017-0257-8

**Published:** 2017-08-24

**Authors:** Huan Yang, Jianxing Li, Gang Long, Shaogang Wang

**Affiliations:** 1 Dartment of Urology, Tongji Hospital,Tongji Medical School, Huazhong University of Science and Technology, Wuhan, 430030 China; 2 Department of Urology, Beijing Tsinghua changgung Hospital, Beijing, China; 3 YouCare Technology Co., Ltd, Wuhan, China

**Keywords:** Integrated rigid and flexible Nephroscope, Percutaneous nephrolithotomy, Ultrasound guidance, Renal staghorn stones, Single nephrostomy tract

## Abstract

**Background:**

Renal staghorn stones are challenging for urologists to ensure maximum stone clearance and minimal morbidity. Percutaneous nephrolithotomy (PCNL) has become the gold standard treatment for renal staghorn stones. To assess the safety and efficacy of a novel integrated rigid and flexible percutaneous nephroscope(Rigi-flex nephroscope) in PCNL for renal staghorn stones.We present our initial experience with this new technique.

**Methods:**

From March to July 2016, a prospective analysis of 3 patients with staghorn stones treated with Rigi-flex nephroscope in PCNLunder totally ultrasound guidance by paravertebral block (PVB) anesthesia was done. PCNL was performed with the rigid section of a 13-Fr Rigi-flex nephroscope firstly and the stones were disintegrated into fragments by holmium laser.Then the stones were removed by active flushout, followed by a search for residual stones in other inaccessible calyces with the flexible section. Finally, the residual stones were disintegrated into small fractions by holmium laser in situ or repositioned with a set of disposable retrieval baskets to pelvic or other accessible areas. The whole procedure was accomplished via only one nephrostomy tract. The operating time, stone-free rates (SFR), postoperative hemoglobin drop, complications, length of hospitalization, were recorded.

**Results:**

The operation time were 89, 62 and 45 min, respectively, the postoperative hemoglobin drop was 1, 0.8 and 0.9 mg/dl, respectively.The postoperative Kidney-Ureter-Bladder (KUB) radiograph of the three patients showed no residual fragment >3 mm. No patients needed blood transfusion and suffered significant complications. The length of hospitalization was 9, 6 and 4 days, respectively. No patient needed multiple tracts PCNL or staged auxiliary measures one month after the operation.

**Conclusions:**

The application of Rigi-flex nephroscope in PCNL under ultrasound guidance for staghorn stones has its unique advantages as monotherapy with increasing procedural stone free rate (SFR) via single nephrostomy tract, hence there is less morbidity as it does not require additional tracts dilation and staged auxiliary procedures combination. However, SFR should also be evaluated at a longer follow-up, particularly for staghorn stone, further large-scale multicenter prospective clinical trial are needed to verify its feasibility.

## Background

Percutaneous nephrolithotomy (PCNL) has become the gold standard treatment for large renal stones and currently is recommended for staghorn stones, as it has stone-free rate three times higher than extracorporeal shock wave lithotripsy (ESWL), along with lower morbidity, shorter length of hospital stayand operating time as well as faster return to work than open stone extraction surgery [1–3].Retrograde intrarenal surgery (RIRS) is becoming popular, due to the advances in flexible ureteroscope and holmium laser lithotripsy. It allows retrograde access to the entire intrarenal collecting system in treating renal stones. However, RIRS has high rates of fiber breakage and lower efficiency for larger stones [4].

Most of staghorn stones were approached with PCNL primarily in accordance with existing techniques, but the large stone burden volume and scattered distribution in various parts of the pelvocalyceal system are challenging for most urologists to ensure maximum stone clearance and minimal morbidity.As a rigid endoscope, conventional nephroscope or semi-rigid ureteroscope can not access the renal calyces situated at an acute angle with the calyx of entry, which may increase needs of multiple tracts PCNL or staged auxiliary measures(PCNL or ESWL or RIRS et al.). Creation of multiple tracts to maneuver into various parts of the pelvocalyceal system, for staghorn stones or migrated stone fragments, increases potential risks of access-related morbidity of the procedure [5]. Staged auxiliary measures often accompanied by more medical expenses, with more instruments and procedures [6]. So the preoperative decision of therapeutic schedule should be made to accurately balance cost-efficacy and safety .

It is necessary to explore a new concept and definition of PCNL.So we proposed that PCNL is redefined beyond a surgical technique as a new requirement for the operation procedure:P-Patient oriented, C-Cost efficient, N-New features, L-Less invasive. The new concept of “PCNL” indicates that: the therapeutic schedule including the selection of surgical technique and instruments shall be individualized based on cost-efficacy and safety. The operation shall be completed by novel less-invasive and high-efficacy instruments. Surgeon’s expertise, experience and skills, as well as the new instruments are of upmost impotantance in the new “PCNL” for precise treatment of complex nephrolithiasis.

**Fig. 1:**
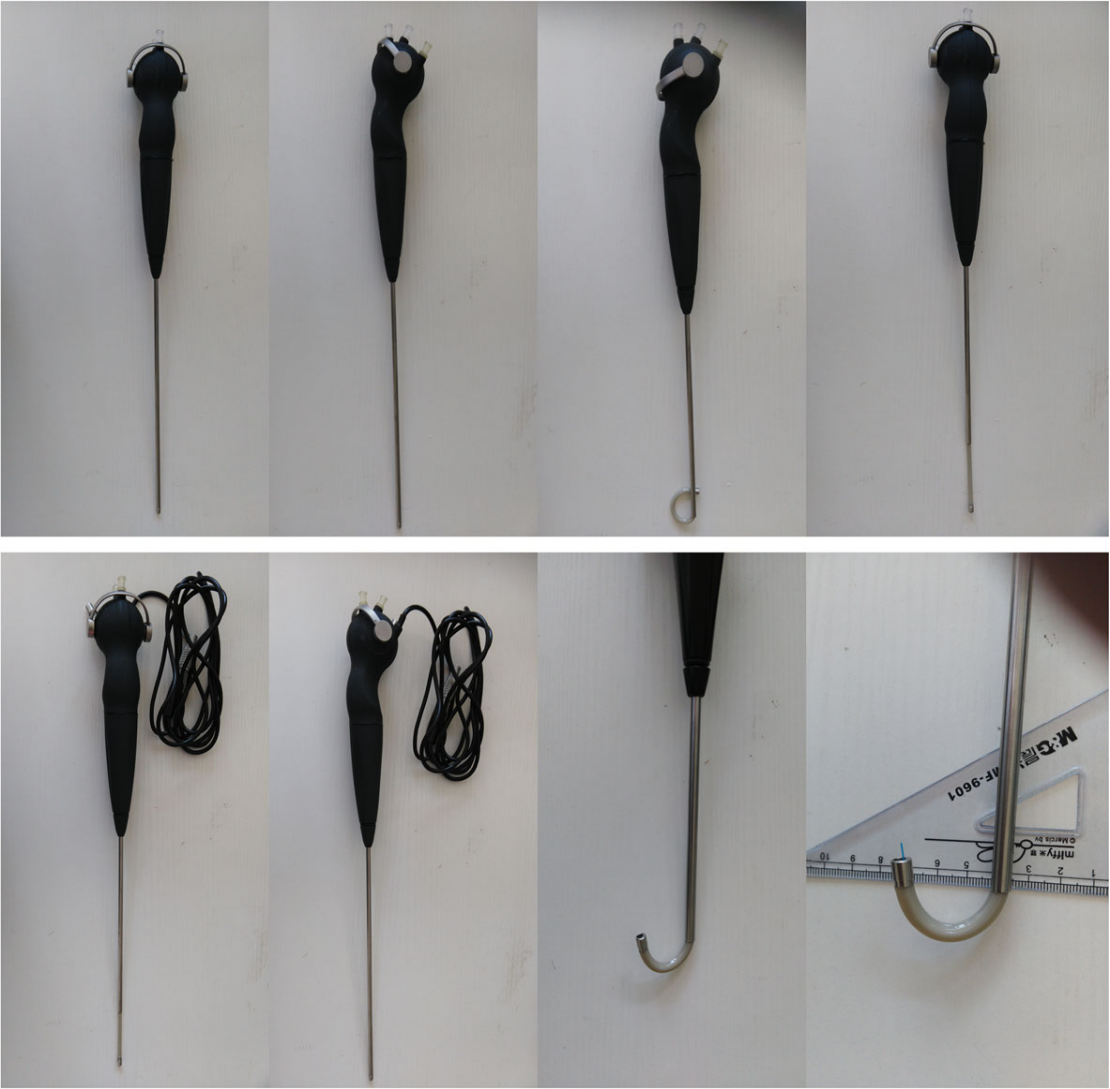
External view of Rigi-flex nephroscope

Based on the new concept of “PCNL”, we proposed “integrated rigid and flexible Percutaneous Nephrolithotomy(Rigi-flex PCNL)” via a novel integrated rigid and flexible nephroscope (Rigi-flex nephroscope) (Youcare, Wuhan, China) for accessing all calyces of the pelvocalyceal system through only one nephrostomy tract, with the objective of assessing the feasibility and safety of this new technique, especially in increasing procedural SFR. The seamless switching of Rigi-flex nephroscope between rigid mode and flexible mode is straightforward. Technical parameters and pictures of Rigi-flex nephroscope are shown in Table 1 and Fig. 1.

**Table 1:** Technical Parameters of Rigi-flex nephroscope

Working length (mm)		
Rigid section	220	
Flexible section	56.5	
Outer diameter (F)		
Rigid section	13	
Flexible section	10.5	
Max deflection angle		
Unloaded	290	
Loaded with 200um Holmium Laser Fiber	270	
Loaded with COOK HWS-035150 Loach Guidewire	210	
Working Channel ID1 (F)	3	
Working Channel ID2 (F)	6	
Unloaded bending times	720	
Minimum bending radius (mm)	10	
Imaging	CMOS	
Using style	One-time use	
Water discharge rate (ml/min)		
	Lower pressure (13.3 kpa)	Higher pressure (26 kpa)
Unloaded working channel	300	400
4.5 F Ultrasonic Probe	120	140
1 F Holmium Laser Fiber	200	300
1.5 F Holmium Laser Fiber	180	220
2 F Holmium Laser Fiber	170	200
COOK HWS-035150 Loach Guidewire	150	188

No reference to a similar study or use of a similar instrument could be found during a thorough literature search. To the best of our knowledge, this is the first clinical study using this endoscope anywhere in the world.

## Methods

Our study was a small-scale clinical observational trial, which used the Rigi-flex nephroscope to evaluate the efficacy and safety of PCNL for patients with staghorn stones. The study was approved by the ethics committees of our hospital. All patients gave written informed consent for the Rigi-flex PCNL and the use of their information in our research, according to the Helsinki II declaration.

From March to July 2016, three patients (two female and one male) with staghorn stones were admitted in our department. Eligible patients were 18 years of age or older with staghorn stones that required PCNL.The demographic characteristics are reported in Table 2. The age of patients was 47, 65 and 53 years, respectively. Two patients had staghorn stones in the right kidney, and one patient had staghorn stones in the left kidney. The maximum diameters of the stones were 4 cm, 3.5 cm and 4.2 cm, respectively.

**Table 2:** The demographics, perioperative data, and complications of patient

Num	Age(years)	Gender	Stone Side	Maximum diameter of stone(cm)	Operation time(min)	Operation time with rigid section (min)	Operation with flexible section (min)	Hospitalization Stay (day)	Postoperative haemoglobin drop rate	Stone free rates(%)	Postoperative recovery time(h)	Complications
1	47	Female	Right	4	89	27	62	9	1	100%	1	fever
2	65	Male	Right	3.5	62	23	39	6	0.8	100%	1	haematuria
3	53	Female	Left	4.2	45	15	30	4	0.9	100%	1	fever

In addition to routine preoperative examination, urine culture and sensitivity was also tested. Preoperative Kidney-ureter-bladder (KUB) radiograph and abdominal non-contrast computed tomography (NCCT) were used to proved staghorn stones and delineate kidney (renal parenchyma, and the distribution in pelvocalyceal system) and adjacent viscera.All patients with urinary tract infection were treated with culture specific antibiotic therapy until repeat urine culture was negative. All other patients with negative urine culture received empiric antibiotic therapy for three days before operation.

After total paravertebral block (PVB) anesthesia was accomplished.An externalized ureteric stent was placed into ureter retrogradely with cystoscope in lithotomy position for retrograde saline injection if intraoperative artificial hydronephrosis is needed, and a 18-Fr Foley catheter was remained in the bladder.Then, the patients were repositioned to prone position with a pillow under the abdomen for establishment of percutaneous nephrostomy tract under totally ultrasound guidance with 3.5-MHz convex abdominal ultrasound probe (BK flex Focus 500, Denmark). The percutaneous renal puncture was finished by an 18G access needle with echogenic tip (Urovision, Germany). First, the surgeons observed the kidney (renal outline and parenchyma, the stone size and distribution in pelvocalyceal system) and adjacent viscera in lower paravertebral region by ultrasound, and then selected an optimum percutaneous puncture spot (The preset spot in skin was pressed by the index finger with low frequency impact from the caudal position behind ultrasound probe, and the shock wave direction of pressed impact wave from the preset spot to the target calyx in ultrasonic imaging plane as the simulative ultrasound-guided needle access was observed and adjusted) usually through the posterior middle calyx, which led straight to maximum stone burden, and kept the adjacent viscera out of the preset needle access. Two-steps precise puncture method was performed for good visualization of entire needle from preset spot into target cylax:First, the echogenic tip of the acccess needle was moved from skin into the perinephric fat tissue where the direction of the needle can be adjusted accompanying respiratory movement.Second, the echogenic tip of needle was inserted into target cylax fornix quickly.

**Fig. 2:**
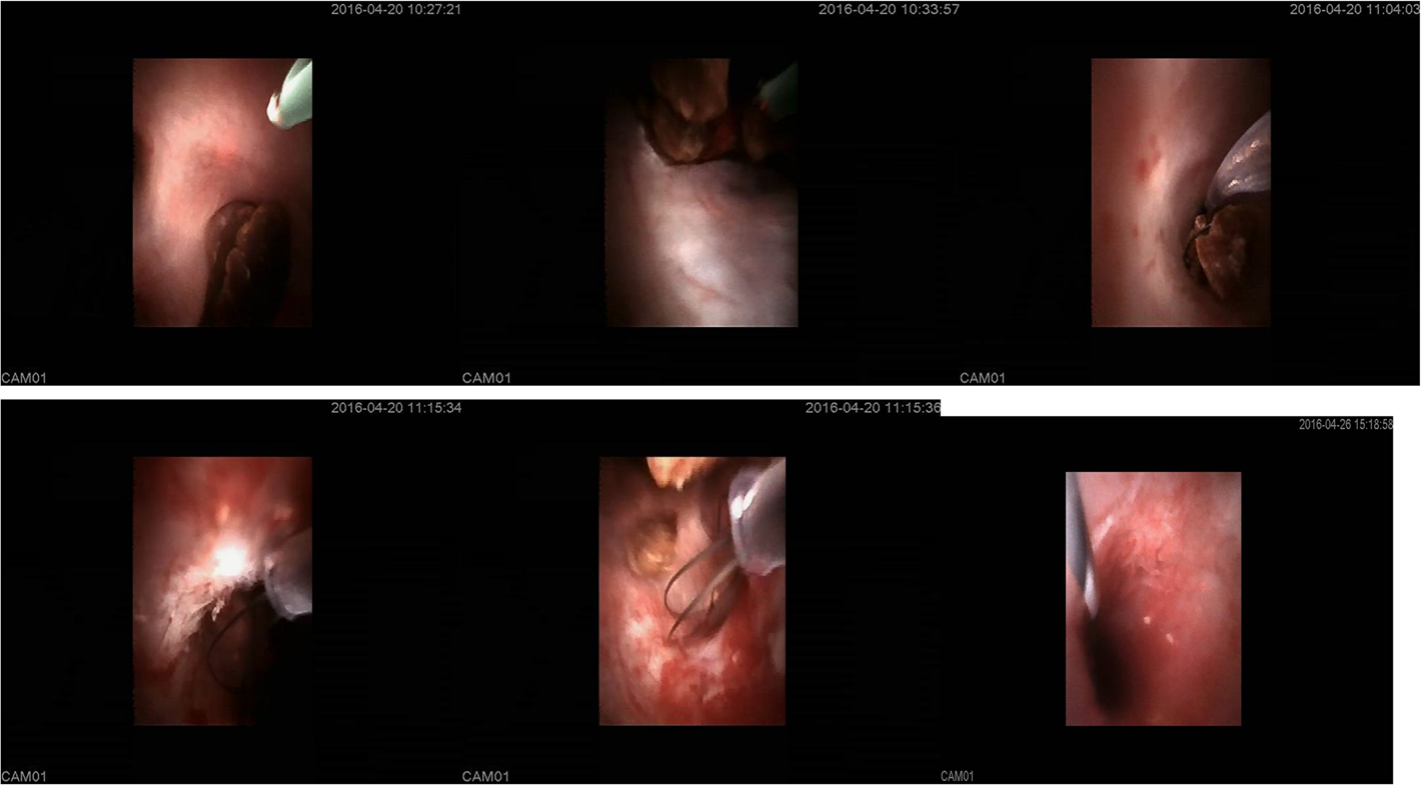
Endoscopic view from Rigi-flex nephroscope demonstrating the digital image quality in operation

If percutaneous renal puncture was accomplished successfully with the flow-out of clear and transparent urine from needle, a hydrophilic 0.035-inch J-tip coaxial guidewire was placed into the collecting system through access needle (ultrasound can detect the J-tip in collecting system). After 1 cm skin surrounding the access needle was incised, tract dilatation was serially performed by 10 to 22-Fr fascia dilators (Cook, USA) through the guidewire and a 22-Fr working access sheath was kept in the collecting system.A 13-Fr Rigi-flex nephroscope was advanced into pelvocalyceal system in its rigid section through working access sheath along the longitudinal axial direction of nephrostomy access. The stones were disintegrated into fragments by holmium laser fiber (PowerSuite 100w, lumenis, USA). A 500-μm laser fiber was used for rigid mode with power setting 3.0/20 Hz.The stones were actively flushed out with transportion of stone fragments by continuous irrigation backflow through access sheath alongside the nephroscope or by filling of collecting system with high pressure and quickly removing the nephroscope resulting in immediate inversion of irrigation water-flow with spillage-like removal of stones via the access sheath [7, 8].All visible accessible stones were broken into fraction and flushed out, then the working access sheath and Rigi-flex nephroscope were pulled back to the renal calyx neck when the rigid section couldn’t access the peripheral calyces situated at an acute angle with the calyx of entry.The flexible section of Rigi-flex nephroscope stretched out and deflected for residual stones and migrated stone fragments, then the stones was broken into fraction with laser in situ or repositioned with a set of disposable retrieval baskets to pelvic or other easily accessible area. A 200-μm laser fiber was used for flexible mode with power setting 1.2 J/20 Hz.The interior pictures of Rigi-flex nephroscope in operation were captured and shown in Fig. 2.

The operations were terminated when no residual fragments could be detected with the help of Rigi-flex nephroscope and ultrasound screening.A double J-tip ureteric stent (Bard, USA) was inserted into ureter antegradly and a 20-Fr nephrostomy tube was placed in each patient.All patients were initially evaluated with KUB in postoperative 1st day.Nephrostomy tubes were removed in postoperative 2nd day.The patients were reevaluated with KUB about one month post-operation and double J-tip ureteric stents were removed. NCCT was performed when the stone status were sub-optimally evaluated with KUB.Residual fragment <3 mm were defined as clinically insignificant residual fragments (CIRF). Larger stones >3 mm were defined as residual stones. Patients who were complete stone free or had only CIRF were considered to have a successful surgery.Stone free rate (SFR), postoperative hemoglobin drop and length of hospitalization was recorded. All complications occurring within one month post-operation were recorded according to the modified Clavien Classification system..

## Results

Theperioperative data and postoperative complications were reported in Table 2 too. No patients complained of pain during operation. Early ambulation could be achieved in 1 h after the operations. The postoperative KUB radiographs of all three patients showed no residual fragment >3 mm.Pre and post-operative radiology for the 3 patients were shown in Fig. 3.2 patients had postoperative fever, which were treated with diclofenac sodium and penicillium carbon alkene respectively. No patients needed blood transfusion and no patients suffered severe complications.The operation time was 89, 62 and 45 min, respectively. The postoperative hemoglobin drop was 1, 0.8 and 0.9 mg/dl, respectively. The lengths of hospitalization were 9, 6 and 4 days, respectively.No patient needed multiple tracts PCNL or staged auxiliary measures (PCNL or ESWL or RIRS) one month after the operation.

**Fig. 3:**
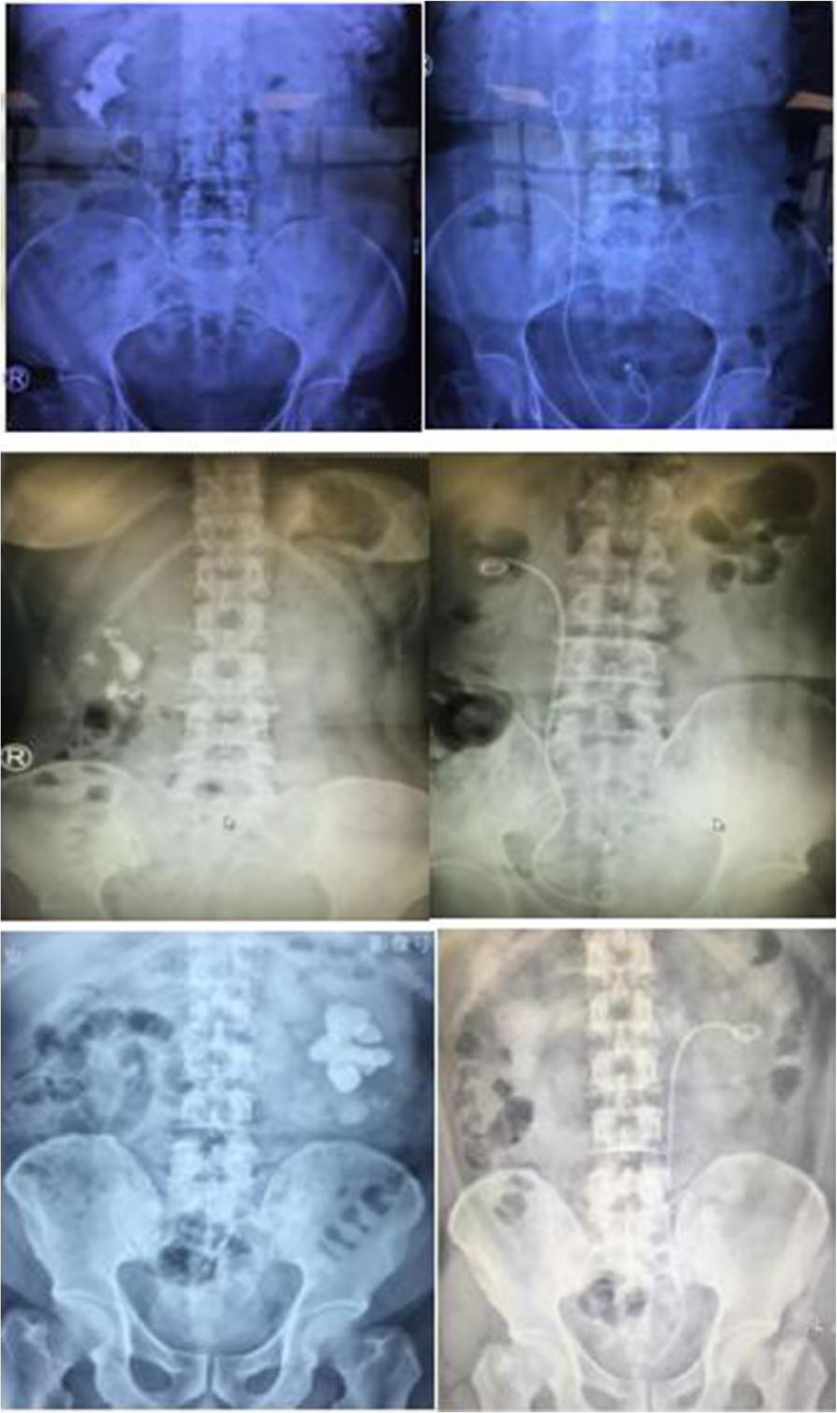
Pre and post-operative radiology for the 3 patients

## Discussion

The first description of percutaneous renal stone extraction was reported by Fernstrom and Johansson in 1976 [9].Wickham and Kollett officially reported and named the Percutaneous Nephrolithotomy (PCNL) in 1981 [10].

The indications for PCNL include stone factors (stone size, stone composition, and stone location), patient factors (habitus and renal anomalies), and failure of other treatment modalities (ESWL and flexible ureteroscopy). The accepted indications for PCNL are stones larger than 20 mm^2^, staghorn and partial staghorn calculi. American Urological Association (AUA) recommends that all newly diagnosed staghorn stones should be actively treated, because untreated staghorn stones have a tendency to destroy the kidney and cause life-threatening urosepsis [2]. It is crucial to completely remove all staghorn calculi, because residual stones can form nuclei for stone recurrence (85% recurrence rate) that may lead to infection [11].

As compared to ESWL as well as RIRS, althrough PCNL with the highest SFR after one-stage single treatment in case of large or multiple renal stones [12, 13].However, It is challenging for the urologist to perform PCNL on staghorn stones and ensure complete stone clearance and minimise morbidity, which may increase the need for multiple tracts PCNL and incidence of staged procedures (Including PCNL, RIRS, ESWL). Conventional PCNL procedure uses a rigid nephroscope or semi-rigid ureteroscope as the level and the neck of access calyx as the pivot, at various angles inside pelvocalyceal system to search for and clear all visible stones in a singe nephrostomy tract. This could cause some certain kidney damage, and even accidental calyx neck avulsion if nephroscope was forced to see the calyx of which the axis form an acute angle with the nephrostomy access for higher stone clearance rate. In severe cases, massive haemorrhage might occur, which blurs the operative vision and hence the procedure needs to be terminated. In such cases, emergency embolization is required, which further increases the medical expense. So to achieve higher complete stone clearance rate for treating multiple stones or staghorn stones, multiple tracts or staged procedure needs to be adopted.

Of the 1466 patients with staghorn stones undergoing PCNL in the CROES database, the SFRs was only 56.9% [14]. SFRs for staghorn stones were even lower in the UK registry [15]. Increasing staghorn volume and complexity may predict the need for multiple tracts and staged procedures for successful stone clearance [16]. Although multiple-tract access did not lead to a more severe reduction in renal function than singe-tract access [17], but multiple-tract access may lead to higher complication rate [18]. Akman et al.demonstrated that >60% of patients with residual stones after PCNL required a second intervention [19]. So PCNL needs continuous improvements for better cost efficacy and less complications.

To the best of our knowledge, this is the first clinical study to use this novel type device-integrated rigid and flexible percutaneous nephroscope in PCNL under totally ultrasound guidance by total PVB anesthesia.As compared with traditional PCNL, Rigi-flex PCNL has its unique advantages:Rigi-flex percutaneous nephroscope can find almost all stones in various calyces with single nephrostomy tract through intraoperative seamless switching of Rigi-flex nephroscope between rigid mode and flexible mode. Medical expenses and access-related morbidity were reduced as it does not require multiple tracts and staged procedures with less instruments. In our initial experience, three patients with staghorn stones underwent Rigi-flex PCNL and were almost completely stone free. The postoperative complications were fewer and minor. The length of hospitalization was slightly longer than before due to treatment of postoperative fever with urosepsis and hematuria. It is a promising tool that has the potential to reduce the morbidity of PCNL in cases of multiple or staghorn stones and improve stone clearance rates.

However, there were certain limitations in our study:This is the initial experience of a new technique from a single center.Since the number of patients included in this study was small, the results were not statistically significant.The SFR should be evaluated in a longer follow-up, particularly for staghorn stones.Further large-scale multicenter prospective clinical trials are needed to verify the cost efficacy and safety of the device.

## Conclusion

The application of Rigi-flex nephroscope in PCNL under ultrasound guidance for staghorn stones has its unique advantages as monotherapy with increasing procedural stone free rate (SFR) via single nephrostomy tract, hence there is less morbidity as it does not require additional tracts dilation and staged auxiliary procedures combination. Given the limited case number, further large-scale multicenter prospective clinical trials are still required.

## Abbreviation

AUA: American Urological Association
CIRF: Clinically insignificant residual fragments
ESWL: Extracorporeal shock wave lithotripsy
KUB: Kidney-Ureter-Bladder
NCCT: Non-contrast computed tomography
PCNL: Percutaneous nephrolithotomy
PVB: Paravertebral block
Rigi-flex nephroscope: Integrated rigid and flexible percutaneous nephroscope
Rigi-flex PCNL: Integrated rigid and flexible Percutaneous Nephrolithotomy
RIRS: Retrograde intrarenal surgery
SFR: Stone free rate
